# Printed Organic Memristive Device on Rigid and Flexible Supports for Neuromorphic Applications

**DOI:** 10.3390/biomimetics11060415

**Published:** 2026-06-11

**Authors:** Davide Vurro, Salvatore Del Basso, Simone Luigi Marasso, Alberto Ballesio, Giuseppe Tarabella, Pasquale D’Angelo, Victor Erokhin

**Affiliations:** 1 Institute of Materials for Electronics and Magnetism, Italian National Research Council (IMEM-CNR), 43124 Parma, Italy; davide.vurro@cnr.it (D.V.); salvatore.delbasso@imem.cnr.it (S.D.B.); simoneluigi.marasso@cnr.it (S.L.M.); alberto.ballesio@polito.it (A.B.); pasquale.dangelo@imem.cnr.it (P.D.); 2 Department of Applied Science and Technology (DISAT), Politecnico di Torino, Corso Duca degli Abruzzi 24, 10129 Torino, Italy

**Keywords:** memristive device, Aerosol jet printing, polyaniline, PEDOT:PSS, flexible devices

## Abstract

Organic memristive devices are promising components for neuromorphic systems. Although based on solution-processable materials, their fabrication often involves complex, resource-intensive processes. Here, we report the fabrication of organic memristive devices using aerosol jet printing to deposit both the active channel based on proprietary polyaniline-based bioink and PEDOT:PSS electrodes. Polymers printing has been carried out both on rigid and flexible substrates, the latter with the aim of demonstrating a flexible device not subjected to films delamination upon bending. By optimizing printing parameters, we achieved devices exhibiting high ON/OFF current ratios exceeding 100 and rapid switching dynamics, with performance comparable on glass and Kapton supports. Morphological and electrical characterizations revealed that channel thickness and uniformity critically influence resistive switching behavior. These findings demonstrate that aerosol jet printing enables scalable, low-material-consumption production of flexible organic memristive devices suitable for neuromorphic applications, potentially facilitating their integration into complex, energy-efficient bio-inspired circuits.

## 1. Introduction

The growing activities in neuromorphic systems, robotics and artificial intelligence are driving the search for new hardware implementations because traditional Complementary Metal-Oxide-Semiconductor (CMOS) electronics is associated with rather high energy consumption. This has stimulated the search for alternative device concepts capable of combining low-power operation with functions closer to those of biological information processing [1]. In this context, memristive devices are promising building blocks for biomimetic information-processing systems [2,3,4,5]. They have been widely employed to emulate synaptic [6,7] and neuronal [8,9,10] functionalities, showing both short- and long-term plasticity and supporting learning rules such as spike-timing-dependent plasticity [11,12], a central mechanism responsible for unsupervised learning in biological systems.

Main efforts on the realization of memristive-based neuromorphic systems are connected to the use of inorganic materials [13,14,15]. This is due to the fact that modern electronics are mainly based on these materials, and this simplifies technological approaches of the fabrication [16].

Nevertheless, organic systems are also seriously considered, due to several important advantages, such as low cost, easy processability, high energy efficiency, low weight, and flexibility [17]. For example, the reached level of energy consumption approaches that of the human brain [18].

Polyaniline (PANI) is by far the first organic material belonging to the class of Organic Mixed Ionic-Electronic Conductors (OMIECs) that was used for the realization of active channels of memristive devices based on a three terminals architecture, i.e., with a silent (reference) gate immersed into a solid/liquid polyelectrolyte interfaced with PANI [19]. These devices have practically all the necessary properties required for neuromorphic systems, allowing their use for the realization of logic with memory, systems with Spike-Time-Dependent Plasticity (STDP) learning [10], reservoir computing systems [20,21] and even as direct synapse analogs upon connecting two live neurons from the rat cortex [22,23].

Despite many advantages, these devices also show limitations, manly connected to the technological aspects concerning their fabrication. In particular, the active channel was fabricated mainly by Langmuir–Schaefer technique, which is time-consuming and difficult to automate [19]. The channel was also fabricated by polyelectrolyte self-assembling [19], but essential characteristics, such as ON/OFF ratio, were established with respect to channels fabricated by Langmuir–Schaefer technique.

In this respect, it seems very promising to use ink-based printing techniques for the devices fabrication. Methods for 3D printed electronics are increasingly becoming popular because of advantages in terms of fast and simple manufacturing while preserving material consumption [24,25]. Printing routes were successfully used for the fabrication of inorganic and organic memristive devices [26,27,28,29,30]. However, only a few works have reported the fabrication of PANI-based memristive devices upon using 3D printing approaches [31,32]. Specifically, it has been demonstrated the appearance of memristive properties in systems where the active channel was formed using 3D printing techniques on test patterns featuring metallic contacts made by MEMS techniques on rigid substrates and the active zone for memristive switching involved PANI interfacing with solid [32,33] or liquid [34,35] electrolytes.

Aimed at investigating the potential of Direct Writing (DW) approaches for rapid prototyping and possible monolithic integration of neuromorphic devices based on flexible/conformable interfaces, the present study investigates the role of the printing parameters on the memristive properties of Flexible Organic Memristive Devices (F-OMDs). F-OMDs were fabricated by Aerosol Jet Printing (AJP) of a PANI aqueous ink [32] defining the active channel between highly conductive Polyethylene Dioxythiophene: Polystyrene Sulfonate (PEDOT:PSS), acting as electrodes, on a polyimide (Kapton) flexible substrate. Special attention is herein paid to ON/OFF ratio and switching time, which will be carefully analyzed and benchmarked against comparable measurements obtained from devices fabricated on rigid glass substrates.

## 2. Materials and Methods

### 2.1. Chitosan-Polyaniline Blended Material Synthesis

This study is based on the use of a chitosan-polyaniline (CPA) blended polymer in the form of a sustainable water-based ink, i.e., free from harmful solvents typically used to prepare PANI suspensions. CPA was indeed synthesized via oxidative polymerization following the protocol proposed by Sajapin et al. [32], leading to a CPA-based ink presenting the polymer in its conducting salt-emeraldine form without the need for film post processing (i.e., soaking in acidic media) [36]. Specifically, 0.2 M hydrochloric acid (HCl) solution of aniline (Merck, St. Louis, MO, USA) was added dropwise, under constant stirring, to a pre-cooled 0.01 M chitosan (Sigma-Aldrich, Burlington, MA, USA) solution prepared in 4% acetic acid (VWR Chemical, Radnor, PA, USA). Subsequently, 0.3 M ammonium persulfate (APS, Acros Organics, Darmstadt, Germany) solution, prepared in 1 M HCl, was added dropwise to the reaction mixture as the oxidizing agent. The resulting solution was stirred continuously and maintained at a temperature below 5 °C for 12 h, during which the color gradually changed from pale yellow to bluish-green and finally to dark green, indicating polymer formation. To quench the excess of APS, 5 mL of a 1 M FeSO_4_ (VWR Chemicals, Radnor, PA, USA) solution was then added. The resulting suspension was centrifuged at 4000 rpm, and the dark green pellet was collected and redispersed in 1 M HCl, followed by another centrifugation. Finally, the solid precipitate was dried in an oven and ground using an agate mortar for future use.

### 2.2. CPA-Based Ink and Printing Parameters Optimization

The CPA was used for ink formulation by dispersing the raw polymer in ultrapure water at a concentration of 25 mg/mL. A 5 mL aliquot of this dispersion was subjected to ball milling using a planetary ball mill (Fritsch Pulverisette, Idar-Oberstein, Germany) in a zirconia jar containing 25 g of 3 mm zirconia spheres. The milling was performed at 450 rpm for a total of 15 h, following a cycle of 15 min milling intervals with 1 min pauses, repeated 60 times. After refinement, the particle size distribution was analyzed via Dynamic Light Scattering (DLS, Nanotrac Flex).

Prior to printing, the refined CPA dispersion was mixed with isopropanol (IPA) in a 4:1 (*v*/*v*) ratio and sonicated for 30 min. The CPA-based ink was deposited using an Aerosol Jet Printer (AJ200, OPTOMEC, Albuquerque, NM, USA) equipped with an ultrasonic atomizer (UA) system. A 2 mL volume of the prepared ink was placed in a UA glass vial and immersed in a thermostatically controlled ultrasonic bath maintained at 20 °C. Before starting deposition, the ink was flowed for 10 min until stabilization.

To optimize printing quality, a preliminary study of the process parameters was conducted, focusing on minimizing overspray, improving line edge definition and control line width and thickness. The parameter that controls the quality of the printed lines is the Focusing ratio (FR) that is equal to the ratio between the Sheat gas flow rate (ShCF) and the Carrier gas flow rate (CGFR). Five different Focusing Ratio (FR) values were tested, with increments of 0.2, across a CGFR flow range of 25 to 35 SCCM in 2 SCCM steps. The printed lines were analyzed using an optical microscope (Nikon Eclypse, Tokyo, Japan) to capture images. Line width and instabilities were then quantitatively evaluated using the open-source software ImageJ 1.54. For statistical analysis, five profiles were evaluated for each printed line. Throughout all experiments, a 200 µm nozzle was employed, with the atomizer current, stage speed, and platen temperature held constant at 0.5 mA, 2 mm/s, and 90 °C, respectively. A sequential characterization of the printing parameters was carried out by keeping the flow rate (FR) constant while varying the printing speed from 1 mm/s to 7 mm/s in 1 mm/s increments. The printed lines were evaluated in terms of their width and thickness using optical imaging (Nikon Eclipse, Tokyo, Japan) and profilometry. Morphology of the layers was studied by SEM (Zeiss Auriga Compact, Oberkochen, Germany).

### 2.3. Devices Fabrication and Electrical Measurements

F-OMDs were fabricated on glass and 50 µm-thick Kapton foil (purchased by RS). Prior the deposition of conductive contacts and CPA layer, the glass and Kapton substrates were cleaned in hot acetone for 10 min, followed by ultraviolet ozone (UV-O_3_) treatment at 50 °C for 6 min. PEDOT:PSS (PH1000 Haeraus) contacts, serving as the source and drain electrodes, were deposited using the AJP technique [37], forming a channel with a W/L ratio of 0.4. PEDOT:PSS was first sonicated for 30 min and filtered through a 0.45 µm syringe filter, then mixed with Ethylene Glycol (EMPLURA) and 3-Glycidyloxypropyl)trimethoxysilane (GOPS, Sigma-Aldrich) at 5 wt% and 1 wt%, respectively. EG-rich formulations have demonstrated high conductivity, in the order of hundreds S/cm [38], hence suitable to define test patterns for CPA-based memristive devices [32].

For the PEDOT:PSS deposition, 2 mL of the ink (prepared with a 1:1 volume ratio of PEDOT:PSS ink to water) was placed in the glass vial of an ultrasonic atomizer. During the printing process, FR, nozzle diameter, stage speed, ultrasonic current, platen temperature, and number of consecutive layers were kept constant at 1.2, 200 µm, 2 mm/s, 0.5 mA, and 2 layers, respectively.

For the CPA deposition, 2 mL of CPA ink (prepared in a 4:1 volume ratio of CPA to IPA) was placed in the glass vial of an ultrasonic atomizer. During the printing process, the FR, nozzle diameter, ultrasonic current, and platen temperature were maintained constant at 1, 200 µm, 0.5 mA, and 90 °C, respectively. Various printing speeds and channel dimensions were tested by adjusting the channel width and length parameters (further details are provided in the Section 3). The thickness as a function of stage speed of the printing lines has been studied through profilometry.

The F-OMD devices were completed by interfacing the CPA channel with a solid polyelectrolyte (SPE) layer composed of polyethylene oxide (PEO, MW 8 MDa, Sigma-Aldrich) doped with LiClO_4_ (Sigma-Aldrich) and AlCl_3_ (Sigma-Aldrich). The SPE gel was prepared by dissolving PEO powder in aqueous solution of LiClO_4_ (0.05 mol/L) and AlCl_3_ (0.1 mol/L) and allowing it to swell under continuous stirring for two days. The resulting gel was then casted onto the CPA channel. Before drying, a silver wire (diameter 0.05 mm purchased from Goodfellow), serving as the silent gate electrode for this special three-terminals architecture [39], was placed perpendicularly to the channel’s length, ensuring no short-circuiting occurred. The assembly was left to dry for 24 h under ambient conditions before electrical measurements.

**Figure 1:**
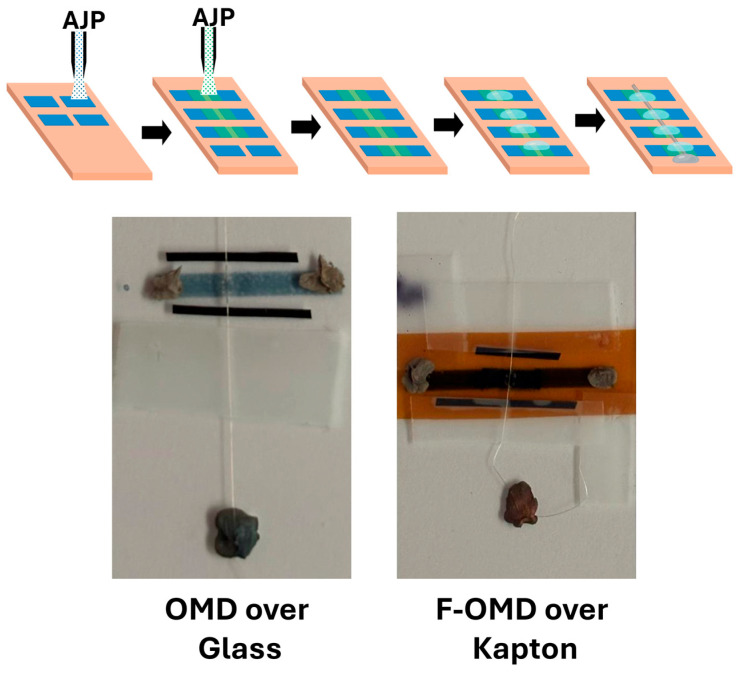
Device assembling procedures. Initially, source and drain electrodes (PEDOT:PSS) are printed (blue regions), followed by the printing of the active CPA channel (green), being both the materials deposited by AJP. A solid electrolyte is cast over the conducting CPA-based channel. Before electrolyte drying, a silver wire (gate) was inserted in it. Photos of fabricated devices are shown in the bottom panel.

The as-described assembling procedure is sketched in Figure 1.

Using a probe station, the as-fabricated devices were connected to a Parameter Analyzer (Keithley 4200A-ScS, Keithley, Cleveland, OH, USA) to carry out the characterization of their electrical response. In detail, source and gate (reference) electrodes were maintained at ground potential, while a voltage ramp was applied to the drain electrode from −0.5 V to 0.7 V (forward scan mode) and reversed (backward scan mode), voltage step ±0.05 V. The current flowing through the device active channel is therefore measured through the hysteresis loop, which is waiting to show the memristive effect. Three switching dynamics consisting of fast (0.1 s), medium (10 s) and slow (30 s) voltage scan rates have been investigated.

The dynamic response has been further analyzed by measuring the resistive switching kinetics; the time variation in the channel current has been accordingly acquired upon switching the device from the ON (biasing at 0.7 V) to the OFF state (biasing at −0.3 V). Both ON/OFF ratio and switching times are obtained by averaging the response of five devices. The Error bars are extracted from device-to-device reproducibility measurements.

## 3. Results and Discussion

### 3.1. AJP Parameters Characterization

**Figure 2:**
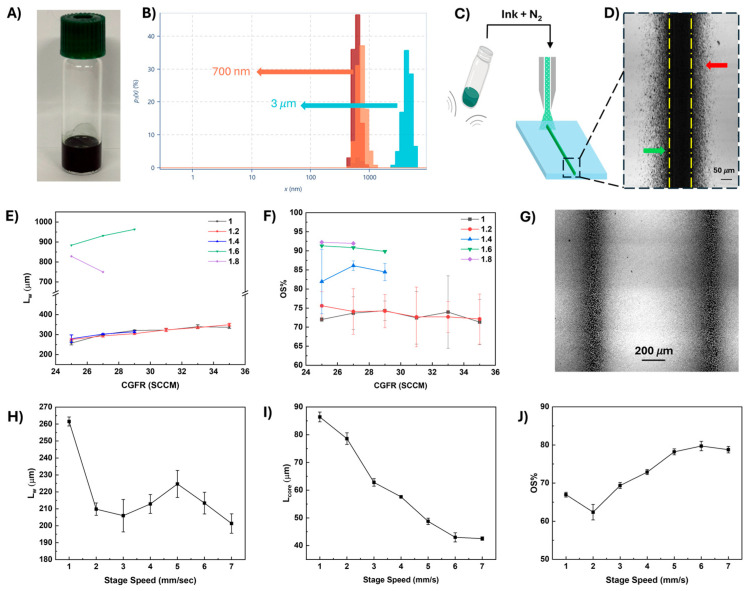
(**A**) Image of CPA ink after ball milling refinement. (**B**) DLS analysis, conducted on CPA ink before (blue) and after refinement (orange), reveals a decrease in size from 3 um to 700 nm. (**C**) Schematic of CPA deposition over glass substrates. (**D**) Image of printing line on glass: red arrow pointed OS defects, green arrow pointed pin holes defects and the part between yellow dotted lines is the L_core_. (**E**) As the CGFR increases, L_w_ initially remains stable at low values. When the flow rate reaches 1.6 and 1.8, the stream exhibits instability, causing L_w_ to become larger than the nominal nozzle diameter (200 µm). Beyond a CGFR of 30 SCCM, clogging phenomena begin to appear. (Error bars for FR 1.6 and 1.8 are not reported due to instabilities that corrupted the printing of 5 different lines). (**F**) The same trend was observed for OS% as a function of CGFR varying FR values. (**G**) Optical image of two narrow lines connected by overspray defects (FR:1.8, CGFR:30 SCCM). Increasing stage speed, (**H**) L_w_ decrease from 1 to 2 mm/sec, from 3 mm/sec up a plateau has observed. (**I**) L_core_ as a function of printing speed and (**J**) OS% as a function of printing speed decrease and increase respectively demonstrating a dependence of printed quality line from stage speed.

Figure 2A shows the image of the final CPA ink dispersion, which remains stable for several months. Herein, the ball milling procedure allowed us to reduce the particle size from approximately 3 µm to about 700 nm (Figure 2B), which improves dispersion stability against sedimentation.

Figure 2C shows the working principle of the AJP system. AJP is a non-contact DW technique able to deposit liquid materials (ink) over a substrate (flexible and/or rigid) with a line resolution of 10 µm [28]. In this process, a liquid ink is placed in a glass vial and atomized using ultrasonic waves, providing that the ink viscosity does not exceed 10 cP.

The resulting ink droplets, typically 1–5 µm in diameter, are carried by a nitrogen gas flow (carrier gas, CG) toward the deposition head. The CG containing the ink droplets is surrounded by a secondary nitrogen flow (sheath gas, ShG), which forms a confining shirt around the aerosol stream to focus the aerosol stream. The focused stream is then directed towards the substrate from the nozzle to allow the ink deposition on a moving stage driven by a CAD. Figure 2D reports an optical image of the CPA ink deposited on a glass substrate. The image reveals a well-defined central region of the printed line (L_core_), surrounded by defects along the edges. These edge defects can be classified into two main types: overspray (OS, red arrow), consisting of small droplets that escape from the deposition cone, and pinholes (indicated by green arrow), which arise from the low wettability of the selected substrate.

To quantify the contribution of these defects to the total line width, we introduced the overspray percentage (OS%), defined as the ratio between the undesired edge region and the total line width Lw [40]. These defects both depend on several factors such as printing parameters, substrates’ surface energy and final ink formulation. Among these, fine optimization of the printing conditions has been selected to minimize them, obtaining a good printing compromise. The first optimization step concerns the relationship between the total Lw and the FR as well as the CGFR. Figure 2E illustrates the dependence of Lw on FR and CGFR. As shown, Lw remains nearly constant following changes in CGFR (i.e., the amount of delivered material). Meanwhile, for lower FR values (1.0, 1.2, and 1.4), similar line widths are obtained. However, at FR = 1.4, printing was interrupted at 29 SCCM due to stream instability. When FR was increased to 1.6 and 1.8, a noticeable increase in Lw was observed. Like 1.4, also for FR equal to 1.6 and 1.8, the deposition falls with increasing CGFR due to the increase in nozzle pressure and related clogging [40,41].

The contribution of printing defects to the final line width was investigated too. Figure 2F shows the OS% as a function of both FR and CGFR. For all tested FR values, OS% exceeded 70% of the total line width, indicating that overspray and pinhole defects significantly influence the final line width. When FR is higher than 1.4, OS% exceeds 90%, highlighting the instability induced by these parameter settings. In contrast, FR values of 1 and 1.2 exhibit OS% values around 75%, suggesting more stable printing conditions. Additionally, no relevant changes in OS% swiping CGFR at FR of 1 and 1.2 have been detected. Also, the printing window is narrowed upon increasing FR values, due to instability enhancement until 1.8, where it is difficult discriminating narrow lines (Figure 2G).

The investigation of the optimal printing outcome was supported by a second characterization step, in which the line features obtained at the optimal FR value of 1 (with ShGFR and CGFR both fixed at 25 SCCM) were analyzed as a function of the printing speed, which is accordingly varied between 1 and 7 mm/s, steps of 1 mm/s. A line width decrease is waited for increased process speed [41]. However, as shown in Figure 2H, the total line width only decreases while passing from 1 to 2 mm/s, remaining almost constant at higher speeds.

To better understand this behavior, a more detailed analysis was carried out by examining L_core_ (Figure 2I) and OS% (Figure 2J) as a function of printing speed. As shown in Figure 2I, the L_core_ values follow the expected theoretical trend, as it decreases with increasing printing speed, reaching a plateau at higher speeds (i.e., 6 mm/s). This behavior is consistent with the fact that, upon exceeding a certain speed, further reduction in line width becomes negligible.

When considering the OS% trend, it is observed that OS% increases with increasing speed. This indicates that while higher speeds reduce the core width, they simultaneously increase the overspray contribution. Consequently, the total printed width slightly increases at higher speeds, as observed in Figure 2H.

Based on the printing quality characterization and considering that the selection of printing parameters relies on a compromise between good quality and stability over prolonged deposition session, the printing parameters optimizing the outcome of CPA deposition by AJP is reported in Table 1.

### 3.2. F-OMD Morphological Characterization

**Figure 3:**
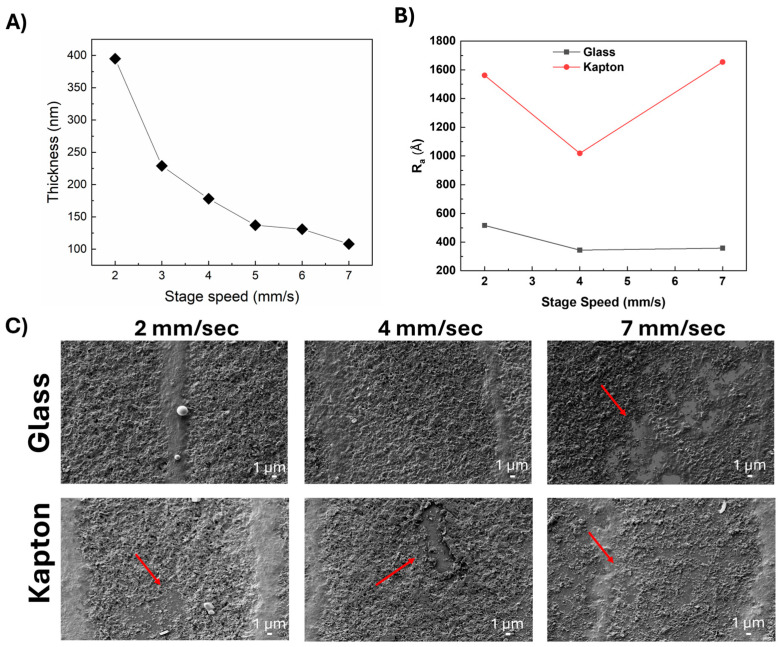
The thickness of the PANI layer (**A**) decreases with increasing stage speed from 1 to 7 mm/s, reaching a plateau at higher speeds (from 5 to 7 mm/s). The surface roughness of the printed CPA is strongly influenced by both the substrate type and the stage speed. The average roughness (R_a_) of the CPA printed on glass and Kapton (**B**) as a function of stage speed (2, 4, and 7 mm/s) reveals a clear substrate dependence. Specifically, the CPA printed on glass exhibits lower roughness compared to that on Kapton, where the substrate’s hydrophobicity dominates the film morphology. This behavior is further corroborated by the SEM images (**C**) of the CPA layers deposited on glass and Kapton at stage speeds of 2, 4, and 7 mm/s. The formation of pinholes and discontinuities (indicated by red arrows) scales with the stage speed and becomes dominant on the Kapton substrate (SEM parameters: Mag = 5.00 kx, EHT = 1 kV, and WD = 3.5 mm).

Figure 3A,B display the profilometry analysis of the printed CPA channel, specifically detailing its thickness and roughness. As expected, the thickness of the printed CPA decreases as the stage speed increases, eventually reaching a plateau at higher speeds where no further reduction in material deposition is observed. Interestingly, surface roughness exhibits a clear dependence on thickness. Figure 3B highlights the average roughness (R_a_) as a function of printing speed for three specific values (2, 4, and 7 mm/s) on both glass and Kapton substrates. For the glass substrate, a reduction in roughness is achieved at higher speeds. Conversely, a more unstable trend is observed for Kapton, suggesting a higher affinity of the ink toward glass than Kapton. Furthermore, the R_a_ values for Kapton are approximately 50% higher than those recorded for glass. These observations are fully consistent with the subsequent SEM analysis.

Figure 3C shows SEM images of the printed CPA layers deposited following the optimized deposition parameters to effectively act as FOMs channels, fabricated on glass and Kapton substrates. The CPA channel was deposited using three different stage speeds: 2, 4, and 7 mm/s. As a result, in case of glass substrates, an increase in the printing speed resulted in a progressive degradation of the layer quality. In particular, higher speeds led to a marked inhomogeneity of the deposited film, as highlighted by the red arrows that indicate the presence of holes and discontinuities.

A completely different behavior is observed when Kapton is used as the substrate. In this case, the substrate properties play a crucial role in film forming. Indeed, Kapton is a hydrophobic and poorly wettable material, and printing on its surface is consistently affected by de-wetting phenomena [40,42]. As a result, discontinuities are already visible at the lowest stage speed of 2 mm/s, but at higher speeds, such as 7 mm/s, layer uniformity is compromised and isolated material clusters not interconnected from each other are observed. This is caused by the hydrophobic nature of the substrate inducing the clustering of the deposited material in localized regions.

### 3.3. F-OMD Electrical Characterization

**Figure 4:**
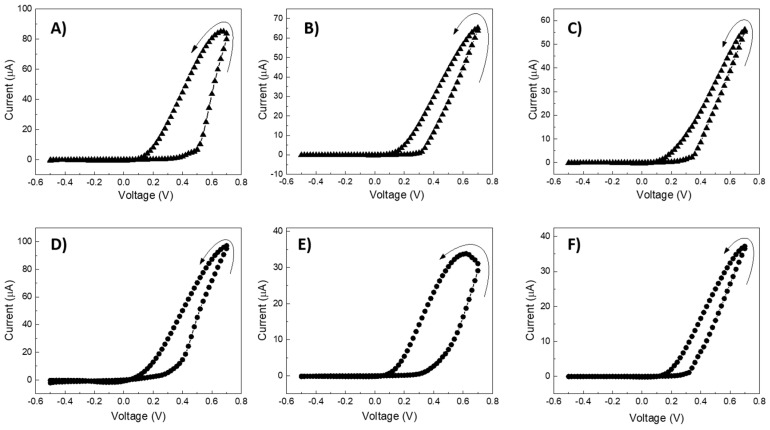
Cyclic voltage-current characteristics for memristive devices fabricated on glass support at process speeds of (**A**) 2 mm/s, (**B**) 3 mm/s and (**C**) 4 mm/s and recorded at a scan voltage rate of 30 s. Arrows indicate the distinctive memristive effect in the hysteresis loop. The same characterization for analogous devices fabricated on Kapton substrates are reported in panels (**D**–**F**), representing the device response for scan voltage rate of 30 s and deposition speed of 2, 3 and 4 mm/s, respectively.

The shape of the cyclic I–V characteristics was found to be dependent on the speed of the voltage scan. Figure 4 shows representative characteristics of devices, fabricated on glass and Kapton supports with the stage speed of 2 mm/s, 3 mm/s and 4 mm/s recorded at voltage scan rate of 30 s between −0.4 V and 0.4 V. In the proposed architecture, Li^+^ ions act similarly to H^+^ to enhance the polymer’s conductivity; however, they require a higher potential of at least 0.4 V (in contrast to the approximately 0.15–0.2 V vs. Ag/AgCl needed for H^+^) [43]. For this reason, 0.4 V was selected as the ON state, while −0.4 V was chosen to record the corresponding current under opposite polarity.

Similar characteristics were observed for devices fabricated at a given deposition speed and characterized at different scan rates; the hysteresis and rectification are more pronounced when the delay between voltage steps is increased, which is typical for OMDs. Although the CPA layer exhibits different morphologies and uniformity when deposited on Glass versus Kapton, their electrical behaviors remain comparable. This phenomenon can be attributed to two main factors. First, the presence of pinholes in these films is not detrimental unlike in PANI films deposited via conventional techniques like Langmuir–Schaefer; instead, here they beneficially increase the active electrochemical interface between the film and the electrolyte [32]. Second, the incorporation of chitosan plays a crucial role. Chitosan is widely utilized as a solid electrolyte in memristor devices due to its inherent ionic conductivity [44], and its presence within the blend enhances the overall ionic transport of the deposited material. Furthermore, its hygroscopic nature promotes a more intimate, continuous contact at the CPA–electrolyte interface compared to the behavior of a dense, uniform layer of pure PANI.

**Figure 5:**
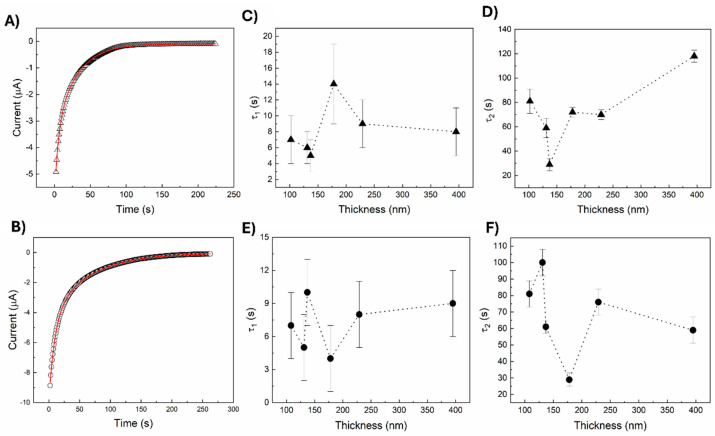
Transient characteristics of the device, fabricated on glass (**A**) and Kapton (**B**) support with the stage speed of 4 mm/s. Dependences of t_1_ (**C**) and t_2_ (**D**) of the devices on glass supports on the thickness of the printed PANI channel. Dependences of t_1_ (**E**) and t_2_ (**F**) of the devices on Kapton supports on the thickness of the printed PANI channel.

To characterize the devices’ resistive switching properties, the transient characteristics have been acquired as follows; first, devices were transferred into the conducting state by the application of a constant channel voltage of +0.6 V for 5 min. After this, they were biased at −0.3 V, then the time variation in the current was registered. The obtained characteristics, acquired for CPA deposited at a stage speed of 4 mm/s, are shown in Figure 5 (panel A for glass supports and panel B for Kapton substrates). Again, devices with channels deposited at different process speeds have demonstrated similar dependences, although providing different dynamics.

Fitting of all registered characteristics was done by using a double exponential decrease (Equation (1)), as follows:

(1) $$I=A_{1}e^{-\frac{t}{\tau_{1}}}+A_{2}e^{-\frac{t}{\tau_{2}}}$$

The choice of a double exponential law characterized by two time constants, namely t_1_ and t_2_, is due to the fact that two processes must be taken into account: the RC contribution of the junction and redox reactions in the polymer bulk, accompanied by field assisted ionic drift between solid electrolyte and CPA channel and back diffusion upon backward voltage scan sweep.

Fitting values of t1 and t2 for the devices fabricated on glass support are reported in Figure 5C,D as a function of CPA thickness, while the analogous characterization for the devices fabricated on Kapton supports are shown in Figure 5E,F.

The first exponent is associated with the RC constant of the system. Accordingly, its fitted value is very similar for all the investigated samples, ranging around 10 s. This exponent is related to the capacitance and resistance between the reference (gate) electrode and CPA channel through the solid electrolyte. Since the electrolyte casting procedure and diameter of the silver wire were kept the same for all samples, with only minor statistical variations, these parameters remain essentially constant across the devices.

In contrast, the second exponent is related to the penetration of ions into and out the CPA channel. This process governs the redox reactions, responsible for the resistive switching behavior. In the case of devices fabricated on glass substrates, the minimum value of t_2_ was observed for the channel thickness of 137 nm, obtained using printing speed of 5 mm/s. As expected, for thicker channels, t_2_ increases, since a longer time is required for ions to penetrate a thicker layer.

The increase in t_2_ observed for thinner layers can be explained as follows. An analysis of the SEM images and the dependence of the channel thickness on the printing speed indicates that thinner layers exhibit poorer homogeneity. In particular, the channel shows an island-like morphology, where some areas are composed mainly of solid electrolyte formed during the device assembling. As a consequence, the potential applied between source and drain electrodes is not uniformly distributed along the channel. Instead, a fraction of the applied voltage is concentrated in regions with a reduced CPA content, which displays higher resistance. This leads to a reduced effective potential difference across the CPA-covered regions, resulting in an increase in the t_2_ value.

For devices assembled on Kapton supports the overall behavior is comparable to that observed on glass. However, a significant difference is present; the minimum t_2_ value is observed for a thicker channel (178 nm). This behavior can be attributed to the fact that, on Kapton, a homogeneous CPA layer can only be achieved at lower printing speeds, which in turn leads to thicker channels.

Another important parameter of memristive devices is ON/OFF ratio. The ON/OFF ratio was defined as the ratio between the current measured at +0.4 V channel biasing in the low-resistance state (ON state), and the current measured at −0.4 V, namely in the high-resistance state (OFF state), respectively. These values were extracted from cyclic I-V characteristics recorded with a time delay of 10 s between consecutive voltage steps.

**Figure 6:**
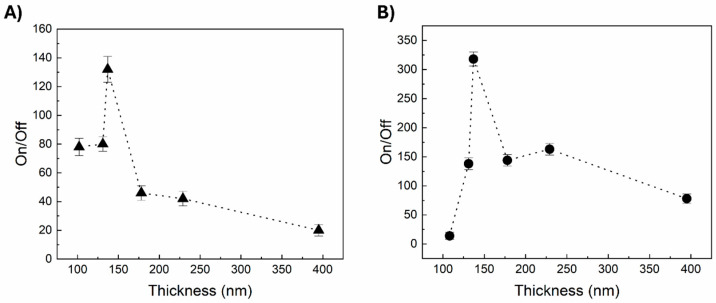
Dependences of ON/OFF ratios on the thickness of CPA channel for devices fabricated on glass (**A**) and Kapton (**B**) supports.

The dependences of the ON/OFF ratios on the thickness of printed CPA channels are shown in Figure 6A,B for devices fabricated on glass and Kapton substrates, respectively.

For both types of devices, the maximum ON/OFF ratio is observed at a CPAPANI channel thickness of approximately 137 nm. The origin of this maximum is directly related to the same mechanisms responsible for the minimum observed in the characteristic time t2t_2t2. At this thickness, the channel exhibits a sufficiently uniform morphology, allowing efficient ion penetration and homogeneous redox reactions throughout the active layer, which maximizes the resistance contrast between the ON and OFF states.

For thicker channels, the ON/OFF ratio decreases because ion transport becomes incomplete within the experimental time window: ions responsible for resistive switching do not reach the deeper regions of the layer, so only a fraction of the channel effectively contributes to the resistance modulation. In contrast, thinner channels suffer from increased structural inhomogeneity, resulting in island-like morphologies with poorly conductive regions. In these areas, both the local electric field and the redox activity are reduced, leading to a less pronounced resistive switching and, consequently, a lower ON/OFF ratio.

Although devices fabricated on Kapton substrates show trends comparable to those observed on glass, substrate-dependent film formation shifts the optimal thickness due to printing-related constraints on layer homogeneity. Overall, these results confirm that device performance is governed by a balance between channel thickness and morphological uniformity, which controls both ion-driven switching kinetics and the achievable ON/OFF contrast. Based on the herein recorded I-V characteristics, power consumption in the ON state ranges around 20 mW, while in the OFF state it approaches values around 20 nanowatt.

The key electrical parameters of memristive devices fabricated on glass and Kapton substrates are summarized in Table 2 and Table 3, respectively.

The reported characteristics allow the identification of optimal printing conditions for the fabrication of CPA channels inf organic memristive devices. Stage speeds in the range of 4–5 mm/s resulted in the highest ON/OFF ratio and the shortest switching times. This printing regime provides the optimal balance between film thickness and morphological homogeneity, enabling redox reactions to occur through the entire layer thickness while simultaneously minimizing regions that are poorly covered by CPA.

## 4. Conclusions

In this work we have fabricated organic CPA-based OMDs using the AJP method, which is the state-of-the-art DW route for 3D printed electronics. Aimed at significantly simplifying the fabrication process of OMDs while assuring a low-material-consumption prototyping, the printing process has been herein implemented to define an all-printed, flexible device endowed with memristive properties, paving the way towards strategies for monolithic integration of OMDs in device networks. The channel electrodes were indeed based on the conducting polymer PEDOT:PSS, which was treated by a well-known secondary doping procedure inducing a strong conductivity enhancement, and a PANI-based sustainable bioink, specifically developed for AJP systems.

The analysis of F-OMDs performances has been carried out in terms of switching capabilities and dynamics, namely by assessing the ON/OFF ratios and the resistive switching time constants As a result, higher ON/OFF ratios registered for CPA-based devices, were found to exceed two orders of magnitude, which is in line with requirements from most of the neuromorphic applications at the state of art, in particular, for the realization of circuits allowing the implementation of the learning function according to bio-plausible algorithms (such as Spike-Timing Dependent Plasticity), as well as artificial neural networks, reservoir computing systems, and other applications at the hardware level. Other promising applications of such devices can be connected to the realization of in-sensor computing systems [45], in which the variation in the output amplitude can be transformed into the variation in spiking sequence, which can be directly analyzed with spiking neuron networks. Regarding the switching dynamics, performance of devices under analysis is in line with that of OMDs based on very thin PANI layers deposited via Langmuir–Schaefer technique and solid polyelectrolytes.

The successful realization of F-OMDs upon exploiting the flexible Kapton as device substrate permits the easy design of new neuromorphic applications taking advantage of the implementation of complex circuits with larger number of memristive devices, which is necessary for mimicking biological benchmarks. The above scenario is largely plausible on the basis of low power consumption shown by the reported elements, which is about 20 mW in the ON state and about 20 nW in the OFF state, compatible with the use of compact batteries for long-lasting power supply.

## Figures and Tables

**Table 1:** CPA ink optimal printing parameters.

Nozzle Diameter (mm)	ShGFR (SCCM)	CGFR (SCCM)	Ultrasonic Current (mA)	Platen Temperature (°C)
200	25	25	0.5	90

**Table 2:** Key parameters for the memristive devices, printed on glass supports.

Speed (mm/s)	Thickness (nm)	ON/OFF Ratio	t_1_	t_2_
2	395	20	8	118
3	229	42	9	70
4	178	46	14	72
5	137	132	5	29
6	131	51	6	59
7	108	78	7	81

**Table 3:** Key parameters for the memristive devices, printed on Kapton supports.

Speed (mm/s)	Thickness (nm)	ON/OFF Ratio	t_1_	t_2_
2	395	78	9	59
3	229	163	8	76
4	178	144	4	29
5	137	318	10	61
6	131	138	5	100
7	108	14	7	81

## Data Availability

The data that support the findings of this study are available from the corresponding author upon reasonable request.
